# Role of Circulating miRNAs as Biomarkers in Idiopathic Pulmonary Arterial Hypertension: Possible Relevance of miR-23a

**DOI:** 10.1155/2015/792846

**Published:** 2015-02-25

**Authors:** Irene Sarrion, Lara Milian, G. Juan, Mercedes Ramon, Idelfonso Furest, Carmen Carda, Julio Cortijo Gimeno, Manuel Mata Roig

**Affiliations:** ^1^Universidad Catolica de Valencia, Guillem de Castro Street 106, 46003 Valencia, Spain; ^2^Department of Pathology, Faculty of Medicine and Odontology, University of Valencia, Blasco Ibáñez Avenue 15, 46010 Valencia, Spain; ^3^Fundación para el Fomento de la Investigación Sanitaria y Biomédica de la Comunitat Valenciana (FISABIO), Cataluña Avenue 21, 46020 Valencia, Spain; ^4^Hospital General Universitario de Valencia, Tres Cruces Avenue, 46014 Valencia, Spain; ^5^Department of Medicine, Faculty of Medicine and Odontology, University of Valencia, Blasco Ibáñez Avenue 15, 46010 Valencia, Spain; ^6^Hospital Universitario Doctor Peset, Gaspar Aguilar Street 90, 46017 Valencia, Spain; ^7^Fundación para la Investigación del Hospital Clínico de la Comunidad Valenciana (INCLIVA), Blasco Ibañez Avenue 15, 46010 Valencia, Spain; ^8^Centro de Investigación Biomédica en Red de Enfermedades Respiratorias (CIBERES), Carretera Soller Km 12, Bunyola, Mallorca, 07110 Illes Balears, Spain; ^9^Department of Pharmacology, Faculty of Medicine and Odontology, University of Valencia, Blasco Ibáñez Avenue 15, 46010 Valencia, Spain; ^10^Fundación de Investigación Hospital General Universitario de Valencia, Tres Cruces Avenue 3, 46014 Valencia, Spain

## Abstract

Idiopathic pulmonary hypertension (IPAH) is a rare disease characterized by a progressive increase in pulmonary vascular resistance leading to heart failure. MicroRNAs (miRNAs) are small noncoding RNAs that control the expression of genes, including some involved in the progression of IPAH, as studied in animals and lung tissue. These molecules circulate freely in the blood and their expression is associated with the progression of different vascular pathologies. Here, we studied the expression profile of circulating miRNAs in 12 well-characterized IPAH patients using microarrays. We found significant changes in 61 miRNAs, of which the expression of miR23a was correlated with the patients' pulmonary function. We also studied the expression profile of circulating messenger RNA (mRNAs) and found that miR23a controlled 17% of the significantly changed mRNA, including PGC1*α*, which was recently associated with the progression of IPAH. Finally we found that silencing of miR23a resulted in an increase of the expression of PGC1*α*, as well as in its well-known regulated genes *CYC*, *SOD*, *NRF2*, and *HO1*. The results point to the utility of circulating miRNA expression as a biomarker of disease progression.

## 1. Introduction

Pulmonary arterial hypertension (PAH) is a disease characterized by a progressive increase in pulmonary vascular resistance, causing an elevation in pulmonary artery blood pressure leading to fatal right heart failure [1, 2]. PAH can be grouped into five categories according to etiology. Idiopathic PAH (IPAH) belongs to group 1 and includes patients with a mean pulmonary artery pressure (MPAP) ≥ 25 mmHg, a pulmonary capillary wedge pressure (PCWP), left atrial pressure, or left ventricular end-diastolic pressure ≤ 15 mmHg, and a pulmonary vascular resistance (PVR) greater than three Wood units [3]. IPAH is a rare disease, with a prevalence of 2-3 per million per year, in which reactive oxygen species- (ROS-) mediated events are important [4, 5]. Recently, the expression of the peroxisome proliferation-activated receptor (PPAR) *γ* coactivator-1*α* (PGC1*α*) has been proposed as a potential biomarker of the progression of IPAH [6]. This protein is involved in the control of the expression of PPAR-regulated genes involved in oxidative metabolism and mitochondrial biogenesis [7].

MicroRNAs (miRNAs) are small (≈22 nucleotides in length) endogenously expressed noncoding RNAs that regulate gene expression at the posttranscriptional level, inhibiting or degrading their target RNAs [8, 9]. These molecules circulate freely in mammalian blood, and several studies have proposed that they can serve as biomarkers for different vascular pathologies, including early myocardial infarction and heart failure in humans [10–13]. miRNAs have been also implicated in the pathophysiological mechanisms involved in the progression of IPAH, including endothelial dysfunction, plexiform lesion formation, smooth muscle cell proliferation, and fibroblast activation and proliferation [14].

Recently, evidence has demonstrated the potential role of circulating miRNAs as biomarkers of the progression of the disease [15]. These studies have examined animal models or isolated lung tissue; to our knowledge, no specific global circulating miRNA expression studies have compared IPAH patients with healthy volunteers.

Therefore, this study used high-density microarrays to study circulating miRNAs in 12 well-characterized IPAH patients. Our results support the potential roles of different miRNAs including the miR23a, miR-130, miR-191, miR-204, miR-145, miR-27a, miR-328, miR-1-2, miR-199, and miR-744 as biomarkers of IPAH. We also highlight the potential relevance of miR-23a because it correlates with the patient's pulmonary function as well as with the potential control of the expression of several circulating mRNAs in which PGC1*α* is included.

## 2. Methods

### 2.1. Study Subjects

This study included 12 well-characterized IPAH patients and ten healthy volunteers. The patient inclusion criteria were MPAP > 25 mmHg, PCWP ≤ 15 mmHg, and PVR > 3 Wood units measured by catheterization. All patients had been treated with different combinations of bosentan, treprostinil, nifedipine, and iloprost before sample collection. The detailed clinical features of the patients, as well as the superoxide dismutase (SOD), PGC1*α*, cytochrome C (CYTC), total antioxidant status (TAS), and glutathione peroxidase (GPX) levels, have been reported [6] and are summarized in Table 1.

All experiments were approved by the Ethics Committee of the University General Hospital of Valencia (Spain) and informed consent was obtained. 5 mL of peripheral blood was extracted from each patient. 2.5 mL of peripheral blood was collected in PAXgene RNA collection tubes (QIAGEN, CA, USA) for total RNA extraction while the serum of the remaining 2.5 mL was reserved for miRNA extraction. Collected samples were stored at −80°C until extraction.

### 2.2. Microarray Analysis

For miRNA global expression analysis, Affymetrix miRNA 2.0 arrays (Affymetrix, CA, USA) were used. miRNA was extracted from serum of the IPAH and healthy volunteers using the miRNeasy Serum/Plasma Kit as recommended by the manufacturer (QIAGEN, CA, USA). Extractions were biotin labeled using the 3DNA Array Detection FlashTag Biotin HSR (Genisphere, Hatfield, USA) and hybridized with the microarrays in a hybridization oven 645 (Affymetrix, CA, USA), following standardized protocols supplied by Affymetrix. Subsequent staining, washing, and scanning of the microarrays were performed using a Fluidic Station 450 and confocal GeneChip scanner 3000 7G (Affymetrix, CA, USA).

Using the GeneChip Command Console Software (AGCC, Affymetrix, CA, USA), DAT and CEL files were acquired. Probe set summarization, background adjustment (using the BC-CG), and quantile normalization were performed using the miRNA QC tool software (Affymetrix, CA, USA). All arrays included in this study met the minimum quality criteria defined by Affymetrix. Extracted data were imported using the dCHIP analysis software (http://www.hsph.harvard.edu/cli/complab/dchip) [16] and statistical analysis was conducted as reported [17].

Global RNA analysis profiles were studied using Affymetrix human genome U133 plus 2.0 arrays (Affymetrix, CA, USA). Total RNA was extracted from the PAXgene RA collection tubes, as described by the manufacturer. Amplification, labeling, hybridization, staining, washing, and scanning of the microarrays followed standardized protocols, with manufacturer-recommended reagents and instruments. DAT and CEL files were acquired using the GeneChip Command Console Software (AGCC, Affymetrix, CA, USA) and the microarrays were analyzed using dCHIP. The invariant set method was used to normalize the data, as reported in [16, 17].

### 2.3. Human Small Vascular Pulmonary Artery Endothelial Cells (HSVPAEC) Culture and miR23a Inhibition

Human small vascular pulmonary artery endothelial cells (HSVPAEC) were isolated and cultured from small vessel of lung as previously described [18]. Segments of pulmonary artery (2–4 mm internal diameter) were dissected free from parenchyma lung tissue, cut longitudinally, and digested with 1% collagenase (Gibco, UK) in RPMI-1640 culture medium for 30 min at 37°C. The digestion was neutralized by adding RPMI-1640 supplemented with 20% fetal calf serum (FCS), and the homogenate was separated by centrifugation at 1100 rpm. The pellet was resuspended, and the cells were cultured in EGM-2 endothelial culture medium supplemented with Single Quotes (Clonetics, UK), 10% FCS, 1% fungizone, and 2% streptomycin/penicillin. HSVPAEC were selected using available Dynabeads CD-31 endothelial cell kit (Dynal Biotech, Germany). The cells were trypsinized (0.25% trypsin) and incubated with CD-31-coated Dynabeads for 30 min at 4°C with end-over-end rotation. After incubation, the HSVPAEC were collected using a magnetic particle concentrator (MCP-1; Dynal) and washed four times with cold phosphate-buffered saline (PBS)/bovine serum albumin (BSA). Purified HSVPAEC retained on the CD-31-coated Dynabeads were separately resuspended in EGM-2 full growth medium supplemented with 10% FCS, 1% fungizone, and 2% streptomycin/penicillin. The cells were grown in 24-well culture plates until 80% confluence. For miR23a inhibiting experiments, the lipofectamine ARNiMAX reagent (Invitrogen, USA) combined with the anti-miR23a MH10644 (Applied Biosystems, CA, USA) was used. Transfection experiments were done following the manufacturer instructions. Efficiency of transfection was evaluated using the GFP coding plasmid supplied by Altogen Biosystems (LA, USA) by fluorescence microscopy.

### 2.4. Real-Time RT-PCR

The relative expression levels of selected miRNAs were studied using real-time RT-PCR using standardized assays on demand (Applied Biosystems, CA, USA) and 10 ng of the same extractions used for microarray analysis. The TaqMan microRNA reverse transcription kit was used for reverse transcription. For each miRNA included in the study we used specific primer supplied by manufacturer (Applied Biosystems, CA, USA). Reactions were performed in a Verity thermocycler (Applied Biosystems, CA, USA) following the manufacturer instructions. For RT-PCR the TaqMan Universal PCR Master Mix No AmpErase UNG (Applied Biosystems, CA, USA) was used with specific assay on demand for each miRNA analyzed. Expression data were obtained using a 7900HT real-time thermocycler (Applied Biosystems, CA, USA) and the relative expression was determined using the semicomparative ΔΔCt method, as reported in [19]. For normalization, the expression levels of miR-374a, miR-374b, and let-7d were studied in the Affymetrix miRNA 2.0 arrays. These three miRNAs have been proposed as endogenous normalizers for serum microRNAs [20]. We found no significant changes in the expression of any of them and miR-374a was selected for normalization because it was the one that exhibited a lower variation across the data.

The relative expression of the* PGC1α*,* CYC*,* SOD*,* NRF2*, and* HO1* genes was studied by real-time RT-PCR as described previously [6]. We used standardized assays on demand (Applied Biosystems, CA, USA) and 100 ng of the same extractions used for total RNA microarray analysis. The cDNA was synthetized using the TaqMan reverse transcription reagents and random hexamers in a Verity thermocycler (Applied Biosystems, CA, USA). RT-PCR was performed in a 7900HT real-time thermocycler (Applied Biosystems, CA, USA) using the 2X Universal Master Mix (Applied Biosystems, CA, USA). The relative expression was determined using the semicomparative ΔΔCt method, as reported in [19]. For normalization, the human GAPDH was selected as housekeeping. The expression of this gene did not change between samples as it was corroborated in the microarrays performed.

### 2.5. Data Analysis

Data are presented as means ± SEM of *n* determinations. Statistical analysis of microarrays was done as described above. For the rest of the determinations, analysis of variance (ANOVA) followed by the Bonferroni test was applied using GraphPad Prism (GraphPad, San Diego, CA, USA). The *t*-test following false discovery rate (FDR) *P* value correction was used to detect significant changes.

## 3. Results

### 3.1. miRNA Profile Changes in IPAH Patients

**Figure 1:**
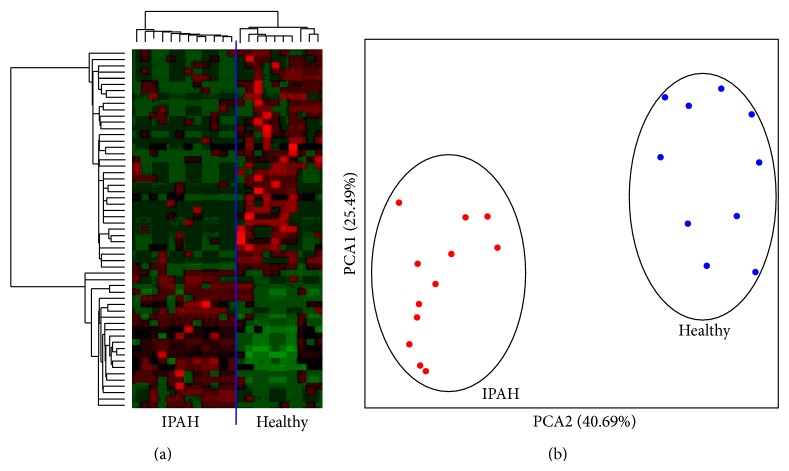
Circulating miRNAs differentially expressed in IPAH patients compared with healthy volunteers. Circulating miRNAs were extracted from blood from 12 IPAH patients and 10 healthy volunteers and analyzed using Affymetrix miRNA arrays. The *t*-test following the false discovery rate (FDR) *P* value correction was used to detect significant changes. Hierarchical clustering (a) and principal component analysis (PCA, (b)) were used to analyze the data obtained.

Circulating miRNA was extracted and analyzed using Affymetrix's microarrays as described above. In this study 12 well-characterized IPAH patients and 10 healthy volunteers were included. *t*-test following false discovery rate (FDR) *P* value correction was used in order to detect significant changes. The results were filtered according to an absolute fold change >2 between the IPAH and healthy groups. We observed significant changes in 61 miRNAs; 36 were downregulated, while 25 were upregulated in the IPAH group compared to the healthy volunteers. Nonsupervised hierarchical clustering and principal component analysis (PCA) were used to classify the data. The results are shown in Figure 1; Table 2 lists all of the miRNA changes.

The data were validated using real-time RT-PCR. Due to the limitation of the sample, representative miRNAs were selected according to their biological significance or degree of change to be validated. We selected 15 miRNas corresponding to near the 25% of the data obtained. All miRNA analyzed by real-time RT-PCR was positively validated. The results are summarized in Table 3.

### 3.2. miR-23a Expression Is Correlated with Pulmonary Function Parameters

Multiple correlation analysis was used to explore the significance of the data. Pearson's correlation was used to compare the relative expression of relevant miRNAs (Table 3) and the value of different parameters indicative of the patients' pulmonary function, including patient age, the 6 min walk test (6MWT), MPAP, cardiac index (CI), and PVR. Of the miRNAs analyzed, significant correlations were found only for miR27a (6MWT), miR199a (6MWT and MPAP), miR744 (PVR), and miR23a (age, MPAP, CI, and PVR) (Table 4).

### 3.3. miR-23a Gene Targets Are Differentially Expressed in IPAH Patients

**Figure 2:**
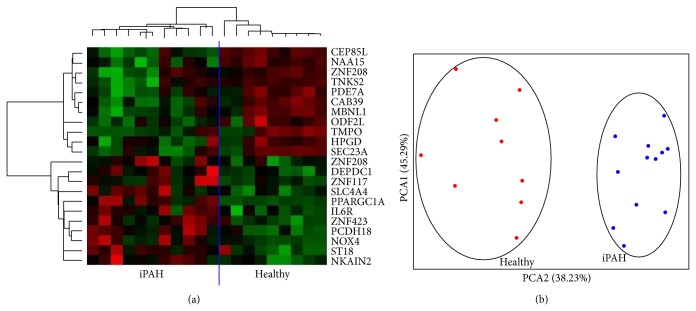
Circulating RNAs differentially expressed in IPAH patients compared with healthy volunteers. Circulating total RNAs were extracted from blood from 12 IPAH patients and 10 healthy volunteers and analyzed using Affymetrix miRNA arrays. The *t*-test following the false discovery rate (FDR) *P* value correction was used to detect significant changes. Hierarchical clustering (a) and principal component analysis (PCA, (b)) were used to analyze the data obtained.

Based on the correlation analysis, we postulated that miR23a might serve as a biomarker of the pulmonary function of IPAH patients. Since miRNAs regulate gene expression, we explored whether miR23a gene targets were differentially expressed in IPAH patients and healthy volunteers. To this end, total RNA was extracted from peripheral blood of the same patients and subjected to global gene expression analysis using Affymetrix microarrays. Significant changes were filtered using ANOVA and FDR correction. The results were filtered according to an absolute fold change between the two groups >2. Genes whose expression is theoretically controlled by miR23a were identified by consulting the miRBase database (http://mirbase.org). Of the 132 genes with significantly changed expression between the IPAH patients and healthy volunteers, 22 (~17%) were potentially controlled at the expression level by miR23a. Interestingly, one of them was* PGC1α*, a gene whose expression was recently proposed as a potential biomarker in IPAH patients [6]. The results are shown in Figure 2 and Table 5.

### 3.4. miR-23a Expression Is Correlated with PGC1*α* mRNA Levels

Finally, we examined the relationship between miR23a and PGC1*α* expression levels using linear regression analysis. We found a significantly negative correlation between the expression of both (*P* = 0.0001, Pearson *r* = −0.7985).

#### 3.4.1. miR23a Inhibition Results in an Elevation of PGC1*α*,* CYC*,* SOD*,* NRF2*, and* HO1* mRNA Expression Levels

**Figure 3:**
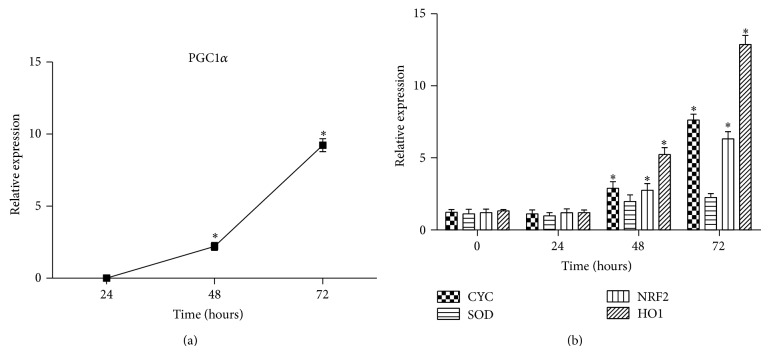
miR23a inhibition results in an increase in the relative expression levels of PGC1*α*. Human small vascular pulmonary artery endothelial cells (HSVPAEC) were cultured and transfected with the anti-miR23a MH10644 for up to 72 hours. Every 24 hours total RNA was extracted and analyzed by real-time RT-PCR for PGC1*α* (a) or for* CYC*,* SOD*,* NRF2*, and* HO1* (b). Three independent experiments were included in each experimental group. Results are represented as mean ± SEM. ^*^ *P* < 0.05 compared to mock transfected cells.

To evaluate the possibility of a direct regulation of PGC1*α* by miR23a siRNA analysis experiments were carried out. HSVPAEC were cultured and miR23a expression was inhibited as described in the Section 2 for up to 72 hours. Every 24 hours total RNA was extracted and relative expression of PGC1*α* as well as its well-known related genes* CYC*,* SOD*, and* NRF2* were analyzed. The results obtained are shown in Figure 3. The relative expression levels of PGC1*α* were increased in anti-miR23a transfected cells compared to mock cultures (Figure 3(a)). This change was in parallel with an increase in the relative expression of* CYC*,* SOD*, and* NRF2* (Figure 3(b)).

## 4. Discussion

This study compared the expression profiles of circulating miRNAs in 12 well-characterized patients and ten healthy volunteers. We found significant changes in 61 miRNAs, as supported by nonsupervised hierarchical clustering and PCA analysis. Different studies have proposed that miRNAs are involved in the regulation of the pathophysiological events leading to the development of PAH.

In this regard, we highlight the relevance of miR204, which is aberrantly expressed in human PAH smooth muscle cells. Its expression correlates with the pulmonary function of PAH patients and its inhibition is related to an increase in smooth muscle cell proliferation and resistance to apoptosis [21, 22]. It was also associated with the protective action of exosomes in a murine model of hypoxic PAH [23]. The miR204 levels were significantly lower in the IPAH subjects compared to the healthy volunteers, supporting the importance of miR204 in the pathogenesis of IPAH [24].

Another important miRNA associated with PH is miR145. Its expression is augmented in patients with heritable PAH and IPAH and BMPR2 mutations increased its expression in mice and PAH patients [25]. We found that miR145 was upregulated in the blood of IPAH patients, supporting its important role in the pathophysiology of PAH. In addition, we found significant changes in miR27a, which is overexpressed in patients with hereditary PAH and is associated with the control of BMPR2-mediated cell proliferation [26], and in miR328, which induces autoapoptosis in smooth muscle cells, inhibiting IGFR1, and acts as a protective agent in PAH [27].

In addition, we observed significant changes in miRNAs related to other pathologies, but not to PAH, including miR-1-2, miR-330, miR-199a, and miR-744. miR-1-2 is involved in the regulation of cardiogenesis, promoting the differentiation of cardiac stem cells into cardiomyocytes; it is also associated with the induction of the antioxidant response of skeletal muscle [28–30]. miR-330 controls the expression of different genes in renal smooth muscle [31]. miR-199a plays an important role in the regulation of HIF1A in cardiomyocytes in hypoxic environments [32]. miR-744 is overexpressed in several cancers, including that of the head and neck [33]. All of these factors are involved in the oxidative stress response, and most regulate the responses of different types of muscle cell, which is in concordance with some of the cellular events involved in PAH.

One of the most interesting miRNAs identified in this study was miR-23a. The overexpression of miR-23a induces cardiac hypertrophy in mice [34]. We found that its expression was increased in IPAH patients compared to the healthy volunteers. There were good correlations with age, MPAP, CI, and PVR, which supports the use of this miRNA as a biomarker of the pulmonary function of IPAH patients. Interestingly, 17% of the genes whose expression levels were changed significantly in IPAH patients compared with the healthy volunteers are theoretically regulated by miR23a. Among these, we highlight PGC1*α*. This protein acts as a coactivator of PPAR*γ* and it was recently proposed to be an important regulator of the antioxidant response of IPAH patients and a biomarker of the pulmonary status of IPAH patients [6]. We found a significant negative correlation between miR23a and PGC1*α* expression, which concurs with recent evidence that miR23a inhibits PGC1*α* expression directly in hepatocellular carcinoma via the IL6-Stat3 signaling pathway [35]. We found upregulated PGC1*α* expression in the IPAH patients compared to the healthy volunteers. This increase in PGC1*α* expression is probably caused by a response to the hypoxia to which these patients are subjected, although this response is insufficient to ameliorate the effects of hypoxia. One of the factors that could be involved in this failure is the abnormal expression of miR23a, which could partially repress the induction of PGC1*α*. To explore this, siRNA experiments have been carried out in cultured human small vasculature pulmonary endothelial cells. Our result indicates that after silencing of miR23a there is a significant increase of the mRNA levels of PPGC1alpha, as well as in the well-known related genes* CYC*,* SOD*,* NRF2*, and* HO1*, which enforces the idea of this relation and is in line with results reported by other authors [36].

Another gene whose expression is controlled by miR23a and was upregulated in the IPAH group is IL6R, which is implicated in the IL6-Stat3 signaling pathway. Monoclonal antibodies that blocked this receptor inhibited breast tumor proliferation and reduced circulating cancer cells, supporting the importance of this pathway in the abnormal proliferation of cells that affect the pulmonary vasculature of IPAH patients [37].

One limitation of this study involves the different treatment supplied to the patients analyzed including different combinations of bosentan, treprostinil, nifedipine, and iloprost. Probably several of the circulating miRNAs involved in the pathogenesis of IPAH are being masked due to the effect of the treatment. More studies including the novo diagnosed patients before and after treatment are needed in order to identify these miRNAs as well as the specific effect of each of these drugs modulating the expression of these masked miRNAs. Data provided here support the usefulness of the analysis of circulating miRNAs as potential biomarkers of the progression of IPAH as well as the possible relevance of miR23a which may be involved in the control of several genes related to the IPAH including PGC1*α*. Nevertheless, more studies are needed to analyze specificity and sensitivity of this molecular tool to be used as biomarker of the IPAH.

## Figures and Tables

**Table 1:** Clinical, molecular, and biochemical features.

ID	Age (years)	Sex	6MWT (m)	PAP (mmHg)	CI (L/min/m^2^)	PVR (WU)	VR	PGC1*α* RE	CYTC RE	SOD RE	TAS (mM)	GPX (nmol/min/mL/)
HP1	45	F	595	48	1.7	12.4	No	2.87	2.45	4.12	0.21	70.23
HP2	75	M	255	70	1.8	11	No	0.34	0.42	0.24	0.05	23.76
HP3	34	F	554	35	2.8	6.2	Yes	67.00	38.50	7.45	0.28	119.05
HP4	58	F	380	48	2.2	12	No	6.07	5.47	2.29	0.19	67.89
HP5	38	F	450	38	3.24	6.1	Yes	8.96	6.95	26.77	0.15	91.27
HP6	63	F	360	40	2.1	8.5	No	3.22	2.59	4.64	0.25	85.91
HP7	64	M	240	53	2.3	7.7	No	0.34	0.48	0.14	0.04	20.22
HP8	66	M	334	50	1.8	8.6	No	1.41	1.01	0.13	0.11	45.42
HP9	60	F	120	49	1.7	18.9	No	0.34	0.08	0.13	0.02	24.26
HP10	55	F	320	31	2.5	7.5	Yes	7.76	8.34	3.47	0.22	90.85
HP11	48	F	534	59	1.9	13.6	No	0.33	0.36	0.17	0.06	19.21
HP12	72	F	280	65	2.2	12.5	No	0.22	0.21	0.50	0.01	14.34

6MWT: 6-minute walk test, PAP: pulmonary arterial pressure, CI: cardiac index, PVR: pulmonary vascular resistance, WU: Wood units, VR: vascular reactivity, PGC1*α*: peroxisome proliferator-activated receptor gamma coactivator-1-alpha, RE: relative mRNA expression, CYTC: cytochrome c, SOD: superoxide dismutase, TAS: total antioxidant status, and GPX: glutathione peroxidase.

**Table 2:** Differentially expressed miRNAs in IPAH patients compared to healthy volunteers (HV).

miRNA ID	Average intensity	Average intensity	Fold change
	(HV)	(IPAH)	
miR-7-1	0.33	1.08	3.25
miR-204	0.78	0.17	−4.67
miR-138-1	1.78	0.50	−3.56
miR-520h	0.78	0.17	−4.67
miR-559	1.11	0.25	−4.44
miR-593	0.67	0.17	−4.00
miR-601	0.56	0.08	−6.67
miR-616	0.89	0.17	−5.33
miR-543	1.11	0.25	−4.44
miR-1184	0.33	1.67	5.00
miR-1285	1.89	6.00	3.18
miR-1286	0.11	0.58	5.25
miR-3153	0.78	0.17	−4.67
miR-3156	0.89	0.17	−5.33
miR-4301	0.78	0.25	−3.11
miR-4304	0.89	0.25	−3.56
miR-4313	1.22	0.33	−3.67
miR-1-2	0.11	0.92	8.25
miR-1259	0.67	0.17	−4.00
miR-1263	0.22	0.83	3.75
miR-193a	0.22	0.75	3.38
miR-195	0.22	0.75	3.38
miR-30c-2	0.67	0.08	−8.00
miR-3120	0.89	0.17	−5.33
miR-3145	0.67	0.17	−4.00
miR-3184	0.33	1.08	3.25
miR-340	0.56	0.08	−6.67
miR-4261	0.78	0.25	−3.11
miR-524	0.56	0.08	−6.67
miR-606	0.89	0.25	−3.56
miR-634	0.56	0.08	−6.67
miR-921	0.56	0.08	−6.67
miR-99a	0.67	0.08	−8.00
miR-181d	0.89	0.17	−5.33
miR-1893	0.89	0.25	−3.56
miR-1934	0.56	0.08	−6.67
miR-1944	0.33	1.08	3.25
miR-1957	0.11	0.83	7.50
miR-1981	0.11	0.75	6.75
miR-20a	0.25	0.79	3.15
miR-145	0.12	0.39	3.21
miR-27a	0.17	0.53	3.12
miR-328	0.89	0.28	−3.15
miR-23a	0.21	0.65	3.09
miR-2145-2	0.22	0.75	3.38
miR-23b	0.33	1.41	4.27
miR-291a	0.56	0.08	−6.67
miR-191	0.11	0.58	5.25
miR-327	0.11	0.58	5.25
miR-423	0.22	0.92	4.13
miR-465b-2	0.22	0.75	3.38
miR-719	0.89	0.25	−3.56
miR-130	0.11	0.83	7.50
miR-124-1	0.11	0.58	5.25
miR-184	0.78	0.25	−3.11
miR-199a	0.67	0.08	−8.00
miR-30e	0.67	0.17	−4.00
miR-330	0.67	0.08	−8.00
miR-362	0.78	0.25	−3.11
miR-513	0.56	0.08	−6.67
miR-666	0.11	0.67	6.00

**Table 3:** RT-PCR validation of differentially expressed miRNAs in IPAH patients compared to healthy volunteers (HV).

miRNA ID	Microarray fold change	RT-PCR fold change
miR-1-2	8.25	12.54
miR-30c-2	−8.00	−5.46
miR-99a	−8.00	−7.25
miR-1957	7.50	10.89
miR-20a	3.15	2.25
miR-145	3.21	3.59
miR-27a	3.12	6.53
miR-328	−3.15	−1.98
miR-23a	3.09	5.59
miR-23b	4.27	6.37
miR-191	5.25	10.27
miR-130	7.50	8.59
miR-199a	−8.00	−12.59
miR-330	−8.00	−5.64
miR-204	−4.67	−3.89

**Table 4:** Multiple correlation analysis of pulmonary function parameters and miRNA expression.

	6MWT (m)		Age (years)		PAP (mmHg)		CI (L/min/m^2^)		PVR (dyn/sec/cm^2^)	
	*P* value	Pearson *r*	*P* value	Pearson *r*	*P* value	Pearson *r*	*P* value	Pearson *r*	*P* value	Pearson *r*
miR27a	**0.0082**	**0.452**	NS		NS		NS		NS	
miR23a	NS		**0.0187**	**−0.44**	**0.0001**	**−0.618**	**0.0055**	**0.607**	**0,0426**	**0.795**
miR199a	**0.0459**	**0.651**	NS		**0.0474**	**0.753**	NS		NS	
miR744	NS		NS		NS		NS		**0.0124**	**0.851**

6MWT: 6-minute walk test, PAP: pulmonary arterial pressure, CI: cardiac index, PVR: pulmonary vascular resistance, and NS: nonsignificant.

**Table 5:** Differentially expressed RNAs in IPAH patients compared to healthy volunteers (HV).

Gene symbol	Average intensity	Average intensity	Fold change
	(HV)	(IPAH)	
CAB39	2.39	0.72	−3.32
SLC4A4	0.88	2.15	2.45
PDE7A	2.59	1.09	−2.38
PPARGC1A	1.36	3.19	2.35
DEPDC1	0.96	3.13	3.26
TMPO	1.91	0.45	−4.21
PCDH18	0.98	2.09	2.13
ZNF208	1.16	2.30	1.98
IL6R	2.15	4.49	2.09
CEP85L	2.51	1.08	−2.31
CENPN	1.01	6.31	6.25
HPGD	1.92	0.84	−2.30
NAA15	2.36	0.71	−3.31
ST18	1.13	2.98	2.64
NKAIN2	1.02	4.32	4.23
SEC23A	2.54	0.78	−3.25
NOX4	1.04	4.25	4.09
ODF2L	1.66	0.18	−9.47
MBNL1	2.77	1.16	−2.39
TNKS2	2.37	0.66	−3.57
ZNF423	1.12	2.78	2.48
ZNF117	1.21	3.04	2.51
